# Supplementary material for: Improving Gene Regulatory Network Inference by Incorporating Rates of Transcriptional Changes
Source: Sci Rep. 2017 Dec 8;7:17244. doi: 10.1038/s41598-017-17143-1 (PMC5722905; doi:10.1038/s41598-017-17143-1)
Supplement: Supplementary file 1 — Supplementary Information [file 41598_2017_17143_MOESM1_ESM.pdf]

Supplemental Figures for "Improving Gene Regulatory Network Inference by Incorporating Rates of Transcriptional Changes"

Jigar S. Desai, Ryan C. Sartor, Lovely Mae Lawas, SV Krishna Jagadish, Colleen J. Doherty

Supplemental Figures

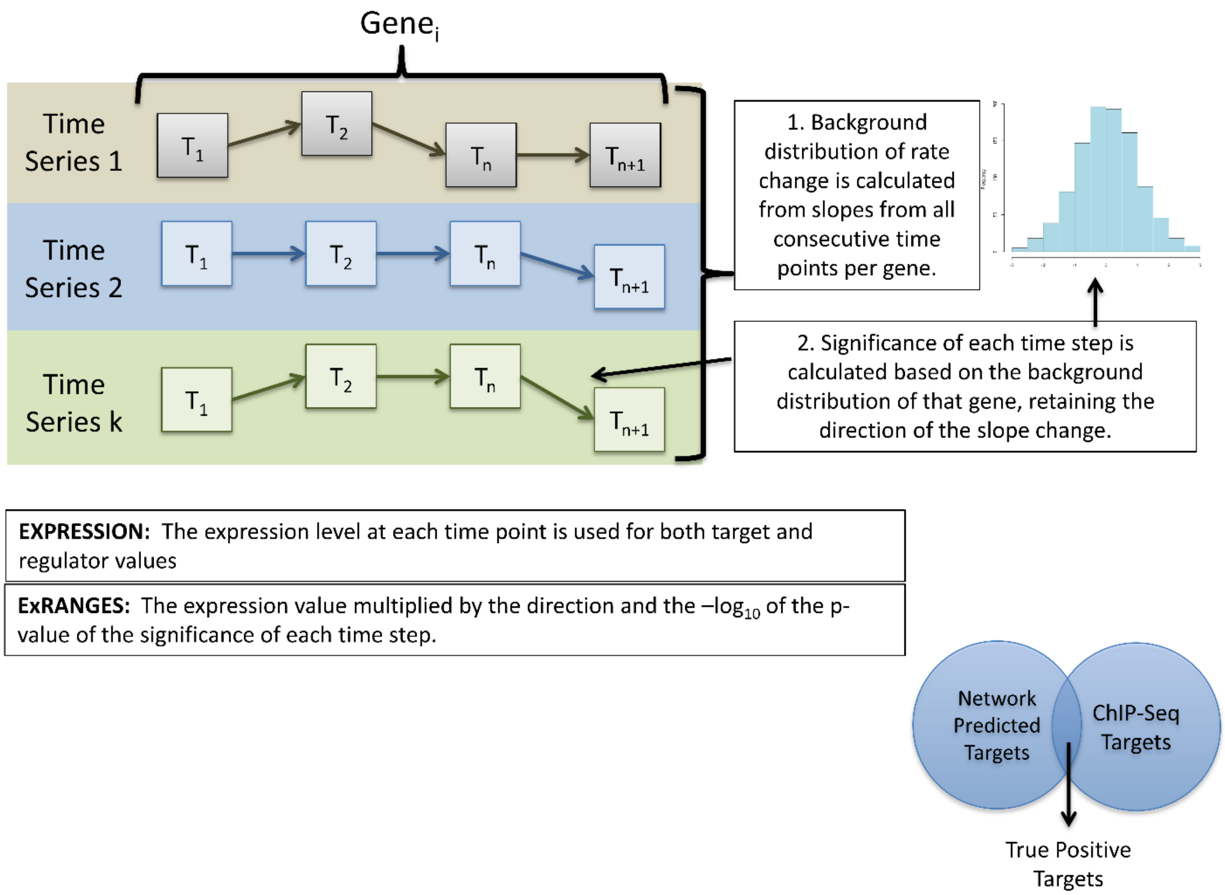

Supplemental Figure 1: Overview of ExRANGES approach. For each Gene$_i$, the slope is calculated for all possible consecutive changes time points. This background distribution is permuted and sampled with replacement and the significance of the change at each time point is calculated. The $-\log_{10}$ of the *p*-value is calculated and the sign change of direction is preserved. In the ExRANGES approach, this significance value is used as the input into network inference using GENIE3 for both the TFs and the targets. For the EXPRESSION approach, the expression values at each time point are provided for both the TF and the targets to GENIE3. The predictive ability of each approach was compared to the targets experimentally identified for each TF by ChIP-Seq or other experimental methods that identify targets of a TF.

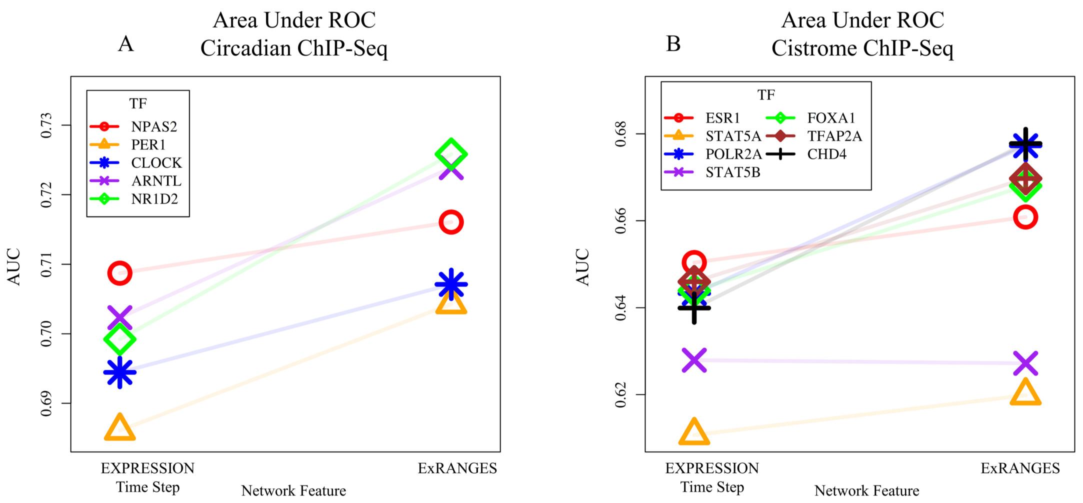

Supplemental Figure 2: ExRANGES outperforms EXPRESSION with added time delay step in identifying targets for select TFs. A) ROC AUC for targets identified with GENIE3 using EXPRESSION with a time delay incorporated between TF and target activation, or GENIE3 using ExRANGES without a time delay step for five circadian TFs. The targets identified computationally were validated against the ChIP-Seq identified targets. B) ROC AUC for targets identified by GENIE3 with an incorporated time delay using EXPRESSION or GENIE3 without the time delay using ExRANGES for seven TFs not known to be components of the circadian clock. Experimentally validated targets for these TFs were identified by ChIP-Seq in epithelial cells, a tissue not included in the expression data [23]**.**

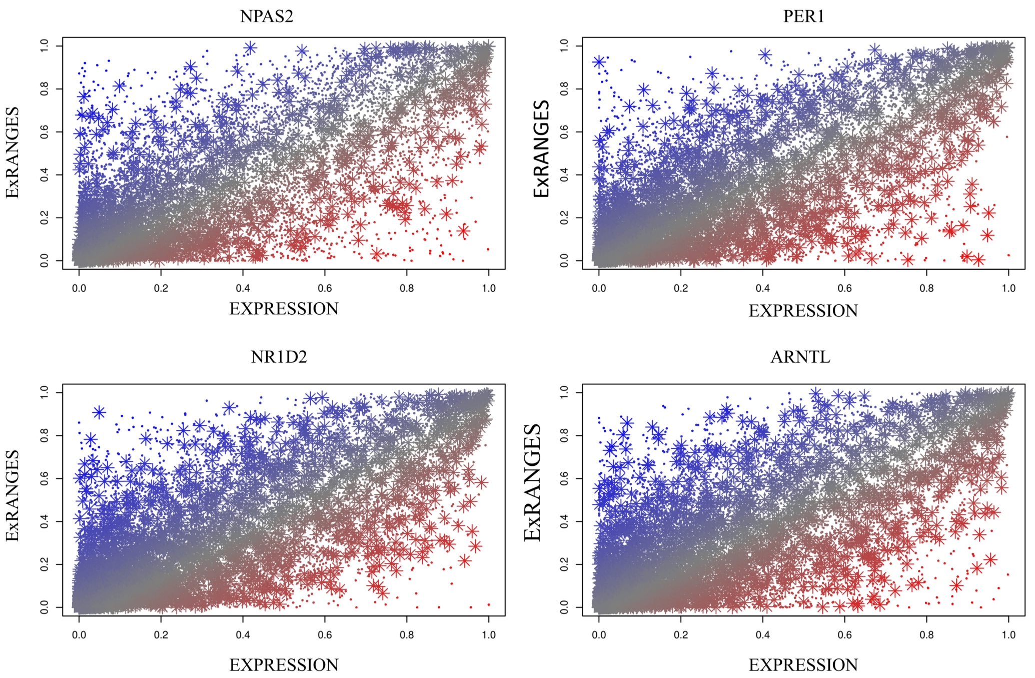

Supplemental Figure 3: TF targets identified differ when using EXPRESSION or ExRANGES as features. Scatter plots showing targets for the TFs A) NPAS2 B) PER1 C) NR1d1 and D) ARNTL. TF targets identified with similar rank by both approaches are shown in grey. Targets identified as high ranking by RANGES are shown in blue and those identified by EXPRESSION are red. TF targets identified by ChIP-Seq [20,21] are marked as stars. Genes that were not in the ChIP-Seq identified targets are plotted as points.

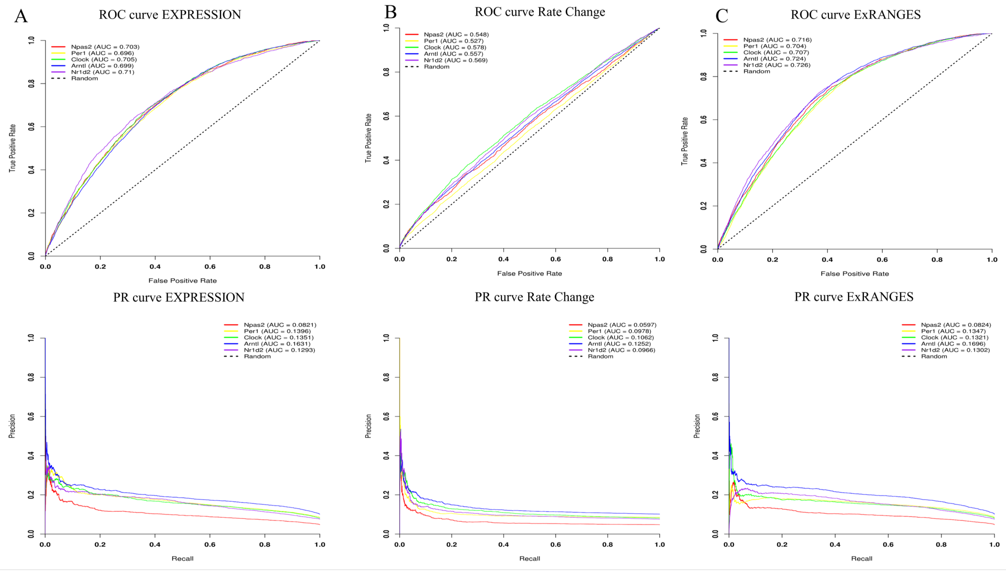

Supplemental Figure 4: Using Rate change alone does not improve identification of TF targets over using EXPRESSION. ROC curves and Precision-Recall curves showing performance on identifying the targets of the five circadian TFs using GENIE3 with A) EXPRESSION as the feature or B) Rate change alone.

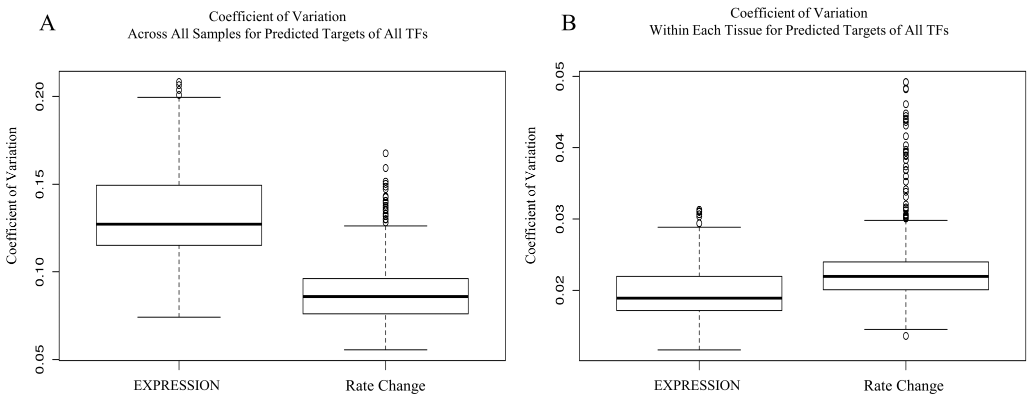

Supplemental Figure 5: Using EXPRESSION alone or Rate Change alone identifies targets with different expression features. A) EXPRESSION identified TF targets show greater variation in expression across all samples. Box plot showing the standard deviation of the top 1000 targets of all TFs identified by either EXPRESSION alone or Rate Change alone. B) Rate change identified TF targets show greater within tissue variation. The standard deviation was calculated for each time series in each tissue for the top 1000 targets of all TFs identified by EXPRESSION or Rate Change. Box plot showing the mean standard deviation for each tissue for these top targets.

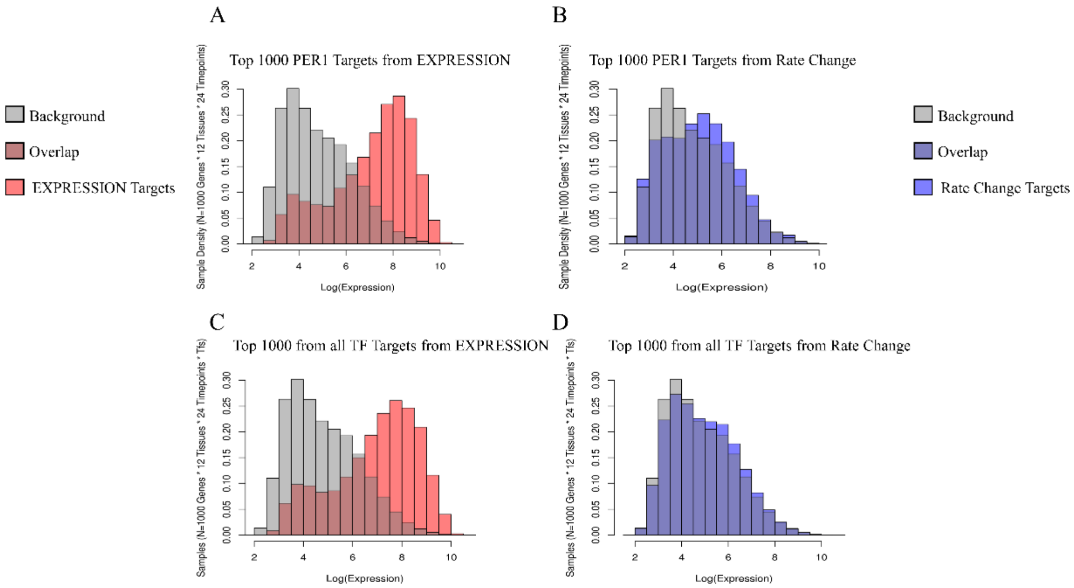

Supplemental Figure 6: Targets identified using EXPRESSION or Rate Change show different distributions of hybridization intensity. Histogram showing the top 1000 PER1 targets identified by A) EXPRESSION (red) have a higher distribution of expression as measured by hybridization intensities compared to the background distribution of all genes (grey). B) Rate Change (blue) identified targets show a similar expression distribution to the background genes. C) The distribution of expression levels of the top 1000 targets identified by EXPRESSION (red) for all TFs is higher than the background gene expression (grey). D) The distribution of expression levels for the top 1000 targets of each TF identified by Rate Change (blue) is similar to the distribution of the expression levels of all genes (grey).

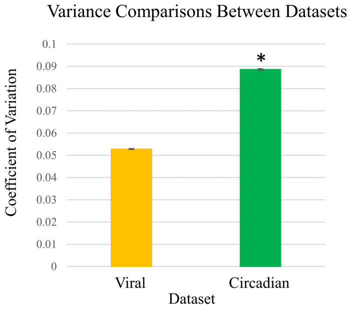

Supplemental Figure 7: Variance comparison of the viral and circadian data sets. The index of dispersion is calculated by dividing the variance of each gene by its mean expression level and taking the mean of these values over all genes in the dataset. The circadian data set showed a significantly higher Index of Variation than the viral data set (Student's t-test, p-value < 10^{-15}).

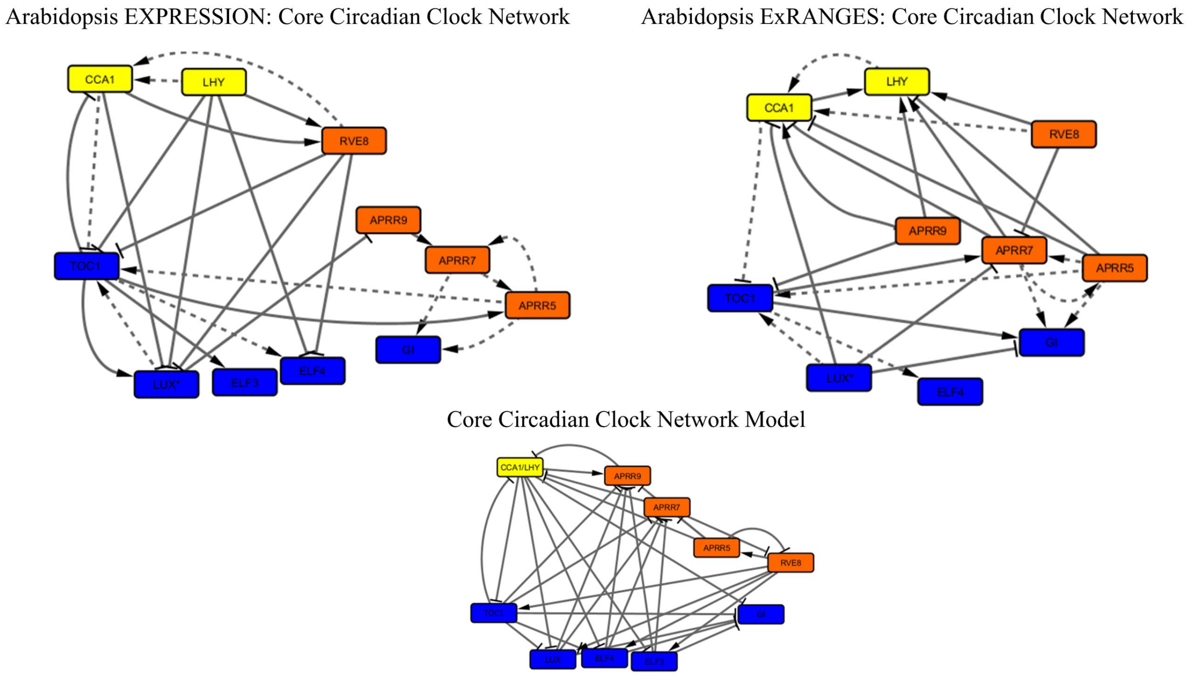

Supplemental Figure 8: ExRANGES improves TF-TF network reconstruction of the Arabidopsis circadian clock. Network model of the TF-TF interactions in the Arabidopsis circadian clock as generated using ExRANGES or EXPRESSION as input to GENIE3. The model from literature as reviewed in Greenham and McClung, 2015 [54] is shown on the bottom for comparison. Each node is colored by the phase of expression: morning (yellow), afternoon (orange), and evening (blue). Dashed edges are predicted interactions that exist in both the EXPRESSION and the ExRANGES networks; solid edges are unique to either the EXPRESSION or the ExRANGES network. The * indicates that the probe set on the microarray corresponding to this gene can bind transcripts from more than one unique locus.

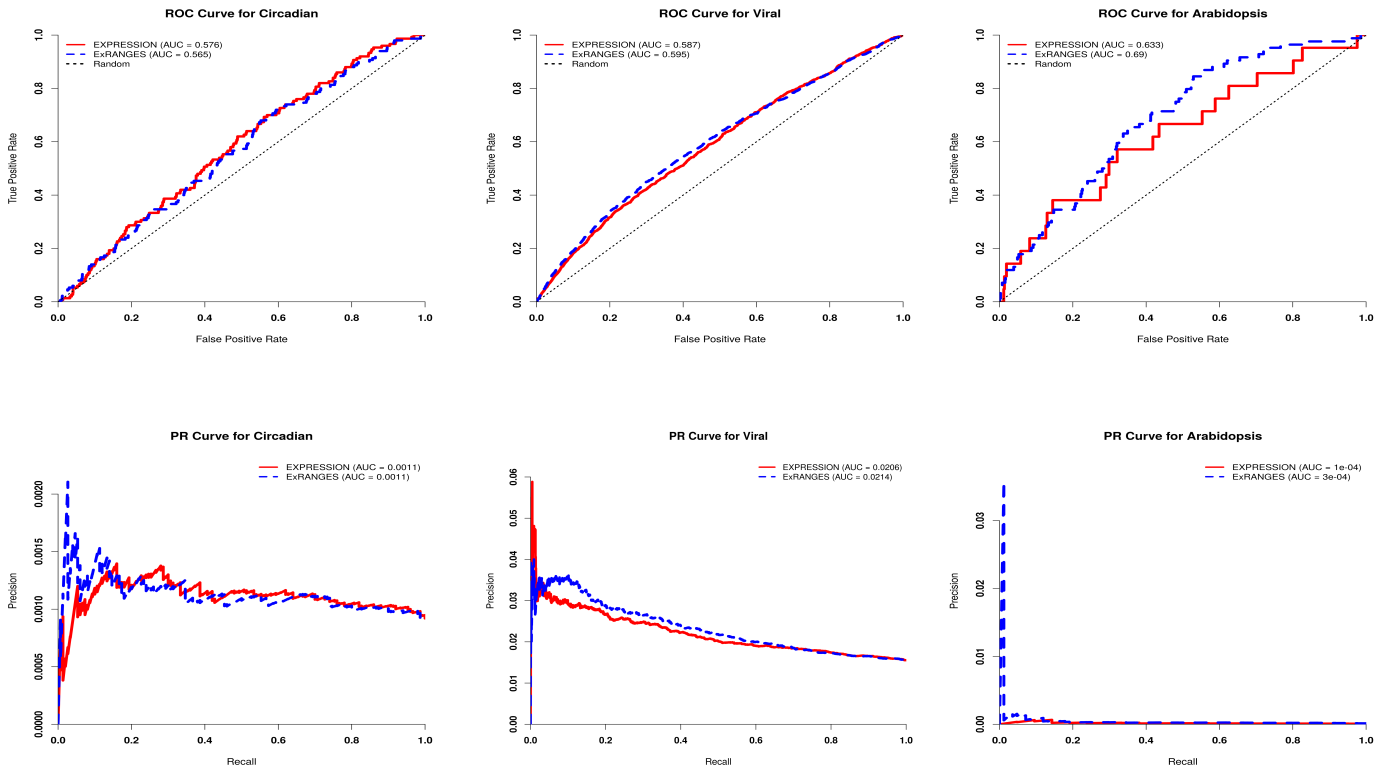

Supplemental Figure 9: Using Inferelator [4], ExRANGES as input improves identification of TF targets compared to EXPRESSION for some data sets. ROC curves and Precision-Recall curves showing performance of Inferelator using EXPRESSION (red, solid) or ExRANGES (blue, dashed) as input. From left to right Circadian is CircaDB data set from mouse tissues; Viral is Human viral data set, and Arabidopsis is Arabidopsis time series data sets across different environmental variables.

	Total In Ref	Ann In Ref	Total In Test	Ann In Test. ExRANGES	Ann In Test. EXPRESSION	Adj_P-Value ExRANGES	Adj_P-Value .EXPRESSION
GO:0005829 - cytosol	11760	2734	1000	334	324	7.79405E-13	9.01398E-11
GO:0005737 - cytoplasm	11760	6677	1000	676	635	4.16632E-12	3.65931E-05
GO:0044403 - symbiosis, encompassing mutualism through parasitism	11760	831	1000	133	123	4.58425E-12	9.73814E-09
GO:0019899 - enzyme binding	11760	1419	1000	187	172	3.03284E-09	9.54676E-06
GO:0007165 - signal transduction	11760	4104	1000	438	420	1.2022E-08	1.06664E-05
GO:0031410 - cytoplasmic vesicle	11760	1334	1000	172	157	8.17064E-08	9.89403E-05
GO:0016192 - vesicle-mediated transport	11760	1126	1000	150	136	9.71356E-08	0.000101302
GO:0005622 - intracellular	11760	7097	1000	685	660	1.58121E-07	0.000403379
GO:0008219 - cell death	11760	1577	1000	194	184	2.22952E-07	3.3731E-05
GO:0043226 - organelle	11760	8381	1000	785	769	3.09897E-07	9.89403E-05
GO:0043234 - protein complex	11760	2273	1000	259	223	6.13509E-07	0.021703931
GO:0005768 - endosome	11760	615	1000	90	67	1.56328E-06	0.050555347
GO:0007155 - cell adhesion	11760	1345	1000	163	154	7.805E-06	0.000403379
GO:0009056 - catabolic process	11760	1512	1000	179	176	9.13287E-06	5.4327E-05
GO:0005764 - lysosome	11760	414	1000	64	54	1.33238E-05	0.005608356
GO:0005575 - cellular_component	11760	10523	1000	934	921	2.32074E-05	0.004835362
GO:0005773 - vacuole	11760	485	1000	71	63	2.33018E-05	0.003275545
GO:0006914 - autophagy	11760	339	1000	54	45	2.87306E-05	0.008123797
GO:0005634 - nucleus	11760	4736	1000	469	443	2.90583E-05	0.012985335
GO:0005794 - Golgi apparatus	11760	1101	1000	134	117	4.55684E-05	0.017414838
GO:0005623 - cell	11760	3174	1000	329	295	4.73443E-05	0.071764584
GO:0003723 - RNA binding	11760	1189	1000	142	124	5.94338E-05	0.022959329
GO:0006810 - transport	11760	3464	1000	354	340	6.13238E-05	0.003727756
GO:0061024 - membrane organization	11760	805	1000	96	95	0.001691272	0.003727756
GO:0006464 - cellular protein modification process	11760	2800	1000	280	275	0.003243441	0.010442251
GO:0008289 - lipid binding	11760	484	1000	61	54	0.004358131	0.056471193
GO:0005783 - endoplasmic reticulum	11760	1185	1000	129	117	0.005621871	0.085457913
GO:0007010 - cytoskeleton organization	11760	911	1000	102	93	0.007203664	0.072551451
GO:0007049 - cell cycle	11760	1347	1000	143	133	0.007765884	0.07181332
GO:0008150 - biological_process	11760	10984	1000	954	949	0.007765884	0.025809124
GO:0065003 - macromolecular complex assembly	11760	1307	1000	139	137	0.008041828	0.015416171
GO:0006461 - protein complex assembly	11760	1018	1000	111	104	0.009864459	0.05957365
GO:0002376 - immune system process	11760	1884	1000	268	277	7.20242E-18	5.57808E-21
GO:0006950 - response to stress	11760	2846	1000	344	359	7.80272E-13	1.12236E-16
GO:0000988 - transcription factor activity, protein binding	11760	496	1000	64	68	0.002144701	0.000425438
GO:0005576 - extracellular region	11760	3265	1000	313	322	0.014987182	0.003727756
GO:0008134 - transcription factor binding	11760	424	1000	49	56	0.04261999	0.003727756
GO:0006412 - translation	11760	445	1000	45	57	0.196057547	0.006084096
GO:0051604 - protein maturation	11760	208	1000	20	31	0.381323161	0.007330201
GO:0030234 - enzyme regulator activity	11760	730	1000	84	85	0.007765884	0.007334146
GO:0007034 - vacuolar transport	11760	85	1000	15	15	0.014934966	0.016803561

Supplemental Table ST1: GO Enrichment for JUND. GO categories enriched in expression in top 1000 JUND targets identified by either EXPRESSION or ExRANGES (FDR adjusted *p*-value <0.01) show more target genes per category in ExRANGES top targets (32 categories) than in EXPRESSION top targets (8 categories). The vacuolar transport category showed no change in the number of the genes annotated in that category in EXPRESSION or ExRANGES targets.

Supplemental Materials and Methods for "Improving Gene Regulatory Network Inference by Incorporating Rates of Transcriptional Changes"

Jigar S. Desai, Ryan C. Sartor, Lovely Mae Lawas, SV Krishna Jagadish, Colleen J. Doherty

Supplemental Materials and Methods

Pseudocode:

LS is a gene by time point matrix with genes as rows and timepoints as columns.
Times is a vector of actual times (usually hours 1 - 24)
To calculate rate of change (RATE values):

```
IF Times Is cyclical THEN
    FOR t=1 to length of LS[1,]
        Rate[,t]=(LS[,t+1]-LS[,t])/(Times[(t+1)]-Times[t])
        IF t=last timepoint THEN
        Rate[,t]=(LS[,1]-LS[,t])/(Times[1]-Times[t])
        END IF
    END FOR
ELSE  FOR t=1 to length of (LS[1,])-1
        Rate[,t]=(LS[,t+1]-LS[,t])/(Times[(t+1)]-Times[t])
    END FOR
END IF
```

Store the sign of each change for use in the final RANGES values:

```
Sign=sign(Rate)
```

Find the Empirical cumulative distribution function for a bootstrapped version of changes for each gene:

```
For g=1 to length of Rate[,1]
    BootstrappedSampled.Rate[g]LS = sample(Rate[g,], n=10,000,
replacement=TRUE)
    Cumulative.Distribution.Functions[g]=ecdf(BootstrappedSampled.Rate[g])
END FOR
```

Calculate P-values using the cumulative distribution function for each gene:

```
For g=1 to length of Rate[,1]
    For t=1 to length of RateLS[1,]
        Pmat[g,t]=Cumulative.Distribution.Functions[g](Rate[g,t])
        IF Pmat[g,t] > 0.5 THEN
            Pmat[g,t]=1-Pmat[g,t]
        END IF
    END FOR
END FOR
```

Log transform and utilize the sign of the change:

```
RANGES = -log10(Pmat)*Sign
```

Multiply RANGES values by the original expression values to get ExRANGES:

```
ExRANGES = RANGES* LS[,(1 to length(Rate[1,]))]
```

Sources for Expression Data Sets

Circadian Data Set

Normalized expression data from murine sources were downloaded from CircaDB [19]. Microarray-based expression levels from 288 samples were used in this study. The data available was from twelve different tissues that were sampled every 2 h for 48 h.

Viral Data Set

The expression data used for the viral experimental analysis was downloaded from GEO GSE73072. This dataset is composed of seven studies of individuals sampled before and after a respiratory infection. The transcript levels are assayed from blood samples of approximately twenty individuals taken over a seven to nine day period, depending on the individual study. Sampling was not evenly spaced between time points. In total, data from 2372 microarrays were used. The expression datasets used for the analyses described in this manuscript were contributed by Drs. Ephraim Tsalik and Geoffrey Ginsburg from Duke University and the Durham VA Medical Center. They were obtained as part of The Respiratory Viral DREAM Challenge through Synapse ID syn5647810 [29,30].

S. cerevisiae RNA-Seq Data

RNA-Seq based expression data from *S. cerevisiae* was downloaded from GEO GSE61668 [33]. This data set was collected from a study to evaluate phosphate starvation in six genotypes of *S. cerevisiae*. Transcript expression was measured by RNA-Seq every 15m for six hours after transfer to reduced phosphate media (150 samples total).

Arabidopsis Circadian Data

Normalized microarray expression data for Arabidopsis were obtained from www.mocklerlab.org/diurnal [36–38,55,56]. This data set consists of transcript data from Arabidopsis plants of various ages grown under 12 different environmental conditions sampled every 4 h for 48 h for a total of 144 samples.

Oryza sativa Diel Data

Rice variety IR64 was grown in the field at the International Rice Research Institute (Manila, Philippines). When the plants reached 50% flowering, panicle tissue was harvested at dawn, dawn + 3.5h, dawn + 7h, dawn + 10.5h, dusk, dawn + 14h, dawn + 17.5h, and dawn + 21h. Four replicates were harvested for each of these eight time points for a total of 32 samples. The third rachis of the panicle was ground in liquid nitrogen with a metal pestle. The tissue was then lyophilized at -60C°overnight. Total RNA was isolated using RNeasy Plant Mini Kit (Qiagen, Germany) with the recommended RLT lysis buffer. The RNA extraction protocol was modified to include an additional incubation with DNaseI. mRNA was isolated from 2 µg of total RNA using magnetic oligo(dT) (NEB, Ipswich, MA). Directional RNA-Seq libraries were prepared from isolated mRNA. Libraries were quantified using a 2100 Bioanalyzer (Agilent, Santa Clara,

CA) RNA-Seq was performed on a HiSeq 2500 (Illumina, San Diego, CA). Reads were trimmed using seqtk (https://github.com/lh3/seqtk). Samples were aligned with Tophat2 to the IRGSP-1.0 genome [57,58]. Counts per gene were identified by HTSeq Count [59].

Selection of Regulators

Transcription factors used as regulators for the murine circadian data and human viral data were obtained from http://www.bioguo.org/AnimalTFDB/index.php [53]. Arabidopsis transcription factor lists were obtained from http://planttfdb.cbi.pku.edu.cn/ [60]. *S. cerevisiae* transcription factors were obtained from [34].

Source for Validation Resources

The direct targets for the five circadian TFs from murine data were obtained from the supplementary information provided in [20]. Targets for additional TFs and the validation of the viral data from human expression data were obtained from the cistrome project (http://cistrome.org/Cistrome/Cistrome_Project.html) [23]. Eighty-three TFs were selected as regulators that were labeled as evaluated blood tissue and present on the HGU133 microarray. ChIP-Seq targets were determined by BETA (http://cistrome.org/BETA/). If multiple ChIP-Seq were provided, the union of both lists was combined. The Arabidopsis ChIP-Seq validations were obtained from multiple sources [40–43,61]. The validation for the yeast analysis was obtained from a TF-DNA binding array from Zhu et al. [34]. The 52 TFs with TF-DNA binding array data were used as regulators and the targets identified by Zhu et al. by the presences of motif sequences with promoter regions of target genes were used to validate the computational approaches.

Detailed Slope and *p*-value Calculation for ExRANGES

The R package ExRANGES has been prepared and is available http://github.com/DohertyLab/ExRANGES. The package performs the following modifications to expression data. *Sample* from the R base package was used to sample 10,000 with replacement for the slopes calculated for each gene. The sampling population is dependent on the time series length (i.e. the circadian data has 48 data points to sample from) *p*-values of the actual slope compared to the distribution of the background slopes were calculated using the Empirical Cumulative Distribution Function, *ecdf,* from the R stat package (R Core Team 2016). See R package provided in http://github.com/DohertyLab/ExRANGES.
